# Morphometic analysis of TCGA glioblastoma multiforme

**DOI:** 10.1186/1471-2105-12-484

**Published:** 2011-12-20

**Authors:** Hang Chang, Gerald V Fontenay, Ju Han, Ge Cong, Frederick L Baehner, Joe W Gray, Paul T Spellman, Bahram Parvin

**Affiliations:** 1 Life Sciences Division, Lawrence Berkeley National Laboratory, Berkeley, California, USA; 2 Department of Pathology, University of California, San Francisco, San Francisco, California, USA; 3 Department of Biomedical Engineering, Oregon Health and Science University, Portland, Oregon, USA

## Abstract

**Background:**

Our goals are to develop a computational histopathology pipeline for characterizing tumor types that are being generated by The Cancer Genome Atlas (TCGA) for genomic association. TCGA is a national collaborative program where different tumor types are being collected, and each tumor is being characterized using a variety of genome-wide platforms. Here, we have developed a tumor-centric analytical pipeline to process tissue sections stained with hematoxylin and eosin (H&E) for visualization and cell-by-cell quantitative analysis. Thus far, analysis is limited to Glioblastoma Multiforme (GBM) and kidney renal clear cell carcinoma tissue sections. The final results are being distributed for subtyping and linking the histology sections to the genomic data.

**Results:**

A computational pipeline has been designed to continuously update a local image database, with limited clinical information, from an NIH repository. Each image is partitioned into blocks, where each cell in the block is characterized through a multidimensional representation (e.g., nuclear size, cellularity). A subset of morphometric indices, representing potential underlying biological processes, can then be selected for subtyping and genomic association. Simultaneously, these subtypes can also be predictive of the outcome as a result of clinical treatments. Using the cellularity index and nuclear size, the computational pipeline has revealed five subtypes, and one subtype, corresponding to the extreme high cellularity, has shown to be a predictor of survival as a result of a more aggressive therapeutic regime. Further association of this subtype with the corresponding gene expression data has identified enrichment of (i) the immune response and AP-1 signaling pathways, and (ii) IFNG, TGFB1, PKC, Cytokine, and MAPK14 hubs.

**Conclusion:**

While subtyping is often performed with genome-wide molecular data, we have shown that it can also be applied to categorizing histology sections. Accordingly, we have identified a subtype that is a predictor of the outcome as a result of a therapeutic regime. Computed representation has become publicly available through our Web site.

## Background

**Figure 1:**
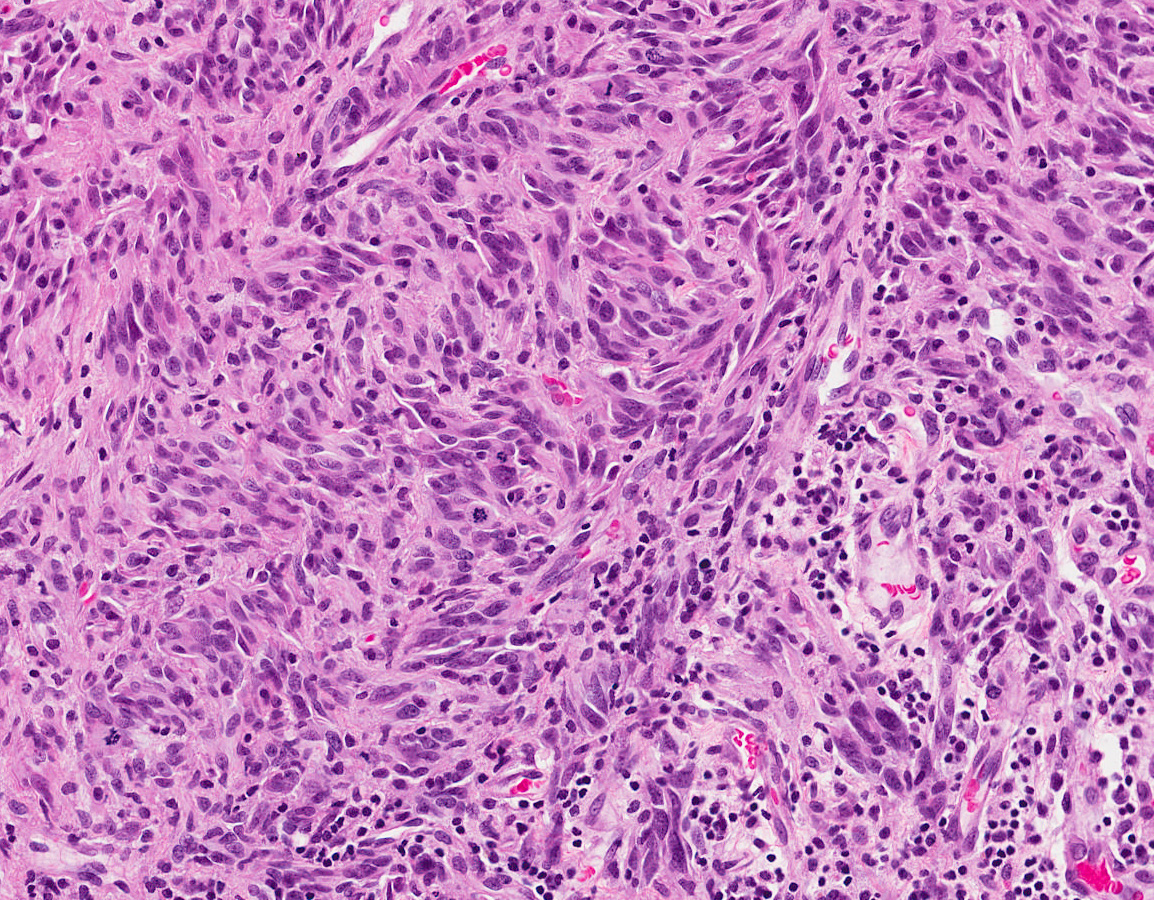
**A pinhole view of GBM tumor section indicates a rich spatial composition in terms of nuclear size, cellularity, and presence of lymphocytes**.

While molecular characterization provides average genome-wide profiling for each biopsy, it fails to reveal inherent heterogeneity that is only visible through tissue histology. Molecular characterization has the advantage of a standardized array-based measurement compared to the genome and other well curated databases. On the other hand, histology sections do not provide standardized measurements, yet they are rich in content and continue to be the gold standard for the assessment of tissue neoplasm. Because of inter- and intra- observer variations [1] and the absence of quantitative representation, some studies have leveraged genome-wide analysis for improved markers for predicting biological behavior. If hematoxylin and eosin (H&E) stained tissue sections can be characterized in terms of cell type (e.g., epithelial, stromal), tumor type, and histopathological descriptors (e.g., tumor specific necrotic rate), then a richer description can be linked with genomic information for an improved basis for diagnostic and therapy. This is the main value of histological imaging since it captures detailed morphometric features on a cell-by-cell basis and their organization. We have tested our system on Glioblastoma Multiforme (GBM), one of the most common and the least curable brain cancer, with glioma cells infiltrating the surrounding tissue with a median survival rate of 14.6 month [2]. Figure 1 shows that the tissue section has a rich spatial composition (e.g., lymphocytes in the lower right side, tumor cells), which is lost through bulk genome-wide array analysis (e.g., microarray, copy number). Our goal is to identify morphometric subtypes, based on nuclear structure and organization, from a very large sample size. First, we provide a brief review of the current state of art and then proceed with the details of our computational strategy. Present techniques for morphometric analysis have focused on several different aspects of tissue characterization, and they are summarized below along with a review of the nuclear segmentation from the H&E sections.

### Brief review of analysis of H&E images

A comprehensive review of techniques for the analysis of the H&E sections is beyond the scope of this paper. However, a brief review can be found in [3]. From our perspective, three key concepts have been introduced to establish the trend and direction of the research community: **(I) **one group of researchers have focused on tumor grading through either accurate or rough nuclear segmentation [4] followed by computing cellular organization [5,6] and classification. In some cases, tumor grading has been associated with recurrence, progression, and invasion carcinoma (e.g., breast DCIS) [7], but such an association is highly dependent on tumor heterogeneity and mixed grading (e.g., presence of more than one grade), which offers significant challenge to the pathologists as mixed grading appears to be present in 50% of patients [8]. A recent study indicates that detailed segmentation and multivariate representation of nuclear features from H&E stained sections can predict DCIS recurrence [9,10] in patients with more than one nuclear grade. In this study, nuclei in the H&E stained samples were manually segmented and a multidimensional representation was computed for differential analysis between the cohorts. The significance of this particular study is that it has been repeated with the same quantitative outcome. In other related studies, image analysis of nuclear features has been found to provide quantitative information that can contribute to diagnosis and prognosis values for carcinoma of the breast [11,12], prostate [13], and colorectal mucosa [14]. **(II) **The second group of researchers have focused on patch-based (e.g., region-based) analysis of tissue sections through means of supervised classification. These methods operate by representing each patch with color and texture features [15,16] for training either a kernel or regression tree classifiers. A recent study evaluated and compared emerging techniques of sparse coding with kernel based methods (e.g., support vector machine, kernel discriminant analysis) on a GBM dataset to conclude that the kernel based method did equally as well, if not better, than sparse coding. Alternatively, some researchers have investigated how architectural features of tumor grades correlate with fractal dimensions [17]. Fractal dimensions differ from topological dimensions and has been shown to have the potential to elucidate irregularities by assigning a gross scalar value for discriminating benign and malignant breast cells from fine needle aspiration [18]. **(III) **A third group of researchers have suggested utilizing the detection of lymphocytes as a prognostic tool for breast cancer [19]. Lymphocytes are part of the adaptive immune response and their presence has been correlated with nodal metastasis and HER2-positive breast cancer, ovarian [20], and GBM. These cells often respond in larger quantity, and they can be easily detected because of their constant size (e.g., approximately 7 micron in diameter) and high chromatin content.

### Brief review of methods for nuclear segmentation

Complexities in delineating nuclear regions originate from both technical (e.g., non-uniform fixation and staining protocol, artifacts in a tissue section, non-uniform thickness in tissue sections) and biological (e.g., different cell types, overlapping compartment) variations. Present techniques have focused on adaptive thresholding followed by morphological operators [21,22], fuzzy clustering [4,23], level set method using gradient information [24-26], color separation followed by optimum thresholding and learning [27,28], hybrid color and texture analysis that are followed by learning and unsupervised clustering [29], and representation of nuclei organization in tissue [30,31] that is computed from either interactive segmentation or a combination of intensity, texture, and morphological operators. Some applications combine the above techniques. For example, in [32], iterative radial voting [33] was used to estimate seeds for the location of the nuclei and subsequently, the model interaction between neighboring nuclei with multiphase level set [34,35]. It is also a common practice that through color decomposition, nuclear regions can be segmented using the same techniques that have been developed for fluorescence microscopy. In recent papers, we [36,37] and others [38] have reviewed those techniques. However, none of these methods can effectively address analytical requirements of the tumor characterization. Thresholding and clustering assume constant chromatin content for the nuclei in the image. In practice, there is a wide variation in chromatin content. In addition, there is the issue with overlapping and clumping of the nuclei, and sometimes, due to the tissue thickness, they cannot be segmented. The method proposed in [32] aims to delineate overlapping nuclei through iterative radial voting [33], but seed detection can fail in the presence of wide variations in the nuclear size; thus, leading to fragmentation. We should also note that many of the techniques that have been developed for analysis of cell culture models, imaged through fluorescence microscopy, are applicable to the analysis of histology sections. Accordingly, methods have been developed to quantify a variety of endpoints using iterative voting [33,39], geometric reasoning [40,41], evolving fronts [35,37,42], and Gabor filter banks [43].

Having summarized the current state of computational histopathology, our objective is to use a large growing dataset of tumor sections and to identify intrinsic subtypes within this dataset. These subtypes can then be used for genomic association. In other words, we don't seek to build a system to mimic histological grading. To meet this objective, it is essential to develop a pipeline for processing a large scale dataset, to overcome technical variations, and to incorporate methods that are extensible to other tumor types. Our testbed consists of 344 sections of GBM, scanned with a 20 × objective in a bright field, which are typically 40,000-by-40,000 pixels.

## Method

### Morphometric analysis and multidimensional profiling

We evaluated a number of nuclear segmentation methods that included level sets [44] or their variants using graph cut implementation, and integration of these methods with seed selection using geometric methods [42]. But these techniques proved to be compute-intensive as a typical tissue section (of size 40k-by-40k pixels) would take roughly a week of processing time on a high end desktop computer. Our experience led to a design of a pipeline that will delineate nuclei and compute morphometric features with a superior computational throughput. The computational model was first validated against synthetic data, then tested on annotated tissue sections, and finally evaluated by a pathologist. Below, we summarize three major components of our methodology.

### Analytical steps

**Figure 2:**
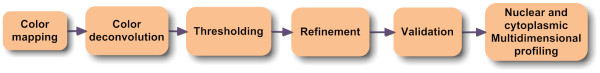
**Steps in delineating each nucleus from an H&E stained tissue sections**.

**Figure 3:**
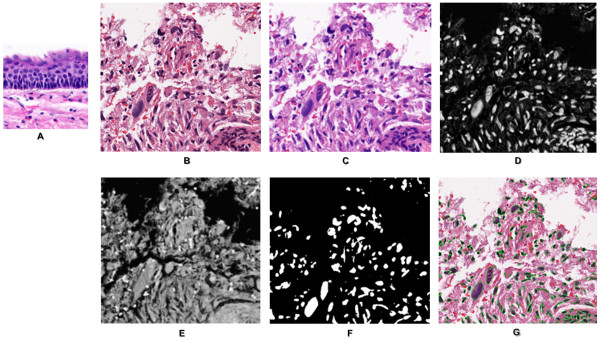
**Steps in delineation of nuclei**. (A) Reference image for color normalization, (B) Original H&E image, (C) normalized image, (D-E) color decomposition for each stain, (F) thresholding, and (G) refinement and validation.

Figures 2 and 3 show steps in converting an image into a multidimensional representation. (I) The first step removes heterogeneity associated with staining by normalizing against one gold standard of H&E stain. (II) The second step performs color decomposition for further reduction of the computational load. The standard approach is a non-negative matrix factorization (NMF) [45], but it is iterative and a previous analysis has indicated NMF did not show superior performance [28]. Here, we used a linear transformation for separating stains [46] based on the orthonormal transformation of the RGB space. (III) The third step computes a threshold from the image corresponding to the nuclei signature. The threshold selection is based on the analysis of the histogram for the value that minimizes intra-class variance. Other techniques, such as modeling foreground and background as two Poisson distributions, yielded similar results. The important issue is fast histogram-based thresholding for subsequent refinement and validation. Refinement consists of enforcement of intensity and geometric constraints. Often, when nuclei are close to each other, either their intercellular contents can leak, the boundaries between the two adjacent nuclei can become perceptual, or the two neighboring nuclei, with completely different chromaticity strength, can merge. The refinement step performs two tasks: (i) it models the intensity distribution of each thresholded blob as a mixture of up to three Gaussians to examine if there is a variation in the background model and whether two adjacent nuclei, with a significantly different amount of DNA content, are merged together, and (ii) it uses the convexity constrain to partition blobs based on perceptual boundaries, as outlined in our earlier paper [40]. Once an image is segmented in terms of nuclear morphology, a multidimensional representation is generated for each nucleus that defines its signature and organization, as we defined in a previous publication [37] and summarized in Additional file 1.

### Computational pipeline

**Figure 4:**
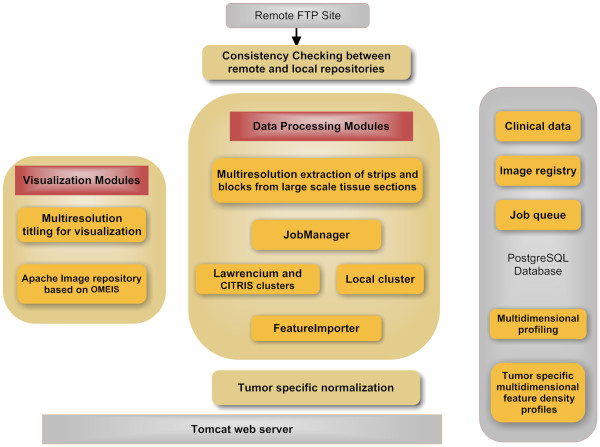
**Computational pipeline consists of four modules: downloads images from the NIH repository**. Each image is partitioned into strips of (1k-by-number of columns), stored in the OMEIS image server. Each strip is partitioned into blocks of 1k-by-1k pixels, where each block is submitted to one of the two clusters at Berkeley Lab. Computed representations are then imported into a PostgreSQL database.

The significance of the pipeline, shown in Figure 4, is that it can process a large amount of data; thus, meeting TCGA data processing requirements. The pipeline has four components: (I) maintaining consistency between the remote and local registries, (II) visualization of tissue sections, (III) data processing and importing computed representation, and (IV) data summarization through normalization.

The pipeline maintains a local registry where consistency between images at TCGA (at the National Cancer Institute) and a local repository is constantly maintained, and new images are downloaded for processing. At present, NCI provides both frozen sections and those from paraffin embedded blocks. Although both types of images are registered and displayed through our system, only paraffin embedded blocks are processed. Each image is partitioned into strips of 1k-by-number of columns, then the strips are stored on the OME image server [47,48].

Visualization of each large scale tissue section is realized through tiling and the utilization of Flash technology that enables a client to pan and zoom, similar to *GoogleMaps™*. Each image (of the order of 40k-by-40k pixels or higher) is partitioned into tiles of 256-by-256 pixels at different resolutions, and the tiles are then stored on a server. As the user drags and zooms on the image in the browser, the tiles are downloaded from the server and inserted into the browser page. Data and images are available through http://tcga.lbl.gov.

Each strip is subsequently partitioned into 1k-by-1k blocks, and blocks are submitted to a computer cluster for processing. The block size has been optimized for processing time and wait time in the queue. At the moment, the entire GBM data set of 344 images takes 4 days of processing. In addition to cluster-based computing, the computational methods of the previous section have a multithread implementation for a more efficient utilization of each computing node. Once each block is processed, computed features are imported into an imaging bioinformatics system, named BioSig [37,49], for further analysis. Several java modules have been developed that run concurrently to access and update the database. The "Jobsubmitter" uses *JSch *(java version of ssh), and *ExpectJ *(java version of Expect) to drive shell scripts on the computing cluster. Computed representation (e.g., nuclear segmentation) can then be overlaid on the original image for quality control.

The backend of BioSig uses PostgreSQL (PG) and summarization of feature-based representation is performed through procedural programming. For high performance applications, PG server programming interface (SPI) enables the transparent transformation of SQL queries. This is a critical component since it adds flexibility for computing morphometric and organization features, normalizing them, and analyzing underlying representation in a new way that was not anticipated. This capability has proven to increase productivity by testing alternative representations without reprocessing the original images. Given the entire GBM (or other tumors) datasets, we have designed a four-step process to normalize each computed feature (e.g., nuclear size, texture, cellularity) for subtyping and genomic association, which is implemented through SPI: (i) each feature is represented as a density distribution per tissue; (ii) feature-based distribution for all tissues, within a tumor type, are combined to construct a global distribution; (iii) the global distribution is then re-binned so that each bin has a similar population of cells of a given feature-value, and (iv) local density distributions are then remapped to computed global bins of equal weight. The net result is that the morphological indices can then be compared, in context, by reporting a distribution function for each feature. These data are downloadable and can be visualized for each tissue section. The rationale for this simplified analysis is that given a large number of cells in a tissue section, classical clustering analysis (for quantization) can be computationally intractable (e.g., computing similarity matrices). In cases where multiple tissue sections exist for a single patient, an average distribution is computed and archived.

### Subtyping and genomic association

Normalized representation of morphometric data are used for subtyping. Subtyping is based on consensus voting [50] by varying the number of subtypes and examining the similarity matrix. It has also been used in earlier papers for subtyping 2D and 3D cell culture morphologies [43,51]. Two gene ranking algorithms of moderated F-statistic and random forests are used for genomic association. (i) Moderated F-statistic [52] utilizes the empirical Bayes method for assessing differential gene expression. In this method, the denominator mean squares (e.g., variance) are moderated across genes through the empirical Bayes approach. The net result is an improved statistical stability given the limited number of samples. The p-value is computed for each gene based on the moderated F-statistic, and then adjusted for multiple hypothesis testing. The adjustment is based on Benjamini and Hochberg's method to estimate the false discovery rate (FDR) [53]. FDR controls the expected proportion of falsely rejected null hypotheses in multiple hypotheses testing to correct for multiple comparisons. The method is implemented through the R Limma package. The top genes that are differentially expressed between subtype 5 and others, with FDR adjusted p-value less than 0.06, are included in Additional file 1 as a heatmap. (ii) Random forest is an ensemble classifier that consists of many decision trees [54]. In random forest, there are several policies for characterizing significance of each gene. One policy evaluates the decrease in classification accuracy by permutation values of a single gene between multiple samples [55]. We used the R implementation of a random forest package [56], where the number of trees (ntree) is increased to 2000 to accommodate the original subset of genes (1740) that were used in an earlier TCGA publication [57]. To insure the robustness and stability of gene selection, the process is repeated by averaging over 100 randomly generated forests.

## Results

The critical factors in our computational pipeline are the throughput, quality of segmentation and morphometric representation for subtyping, and genomic association. The throughput is significant since images need to be continuously processed with a newer version of the software with increased robustness. Presently, the total computational time for 344 large scale tissue sections (from 133 patients) is less than a week on a shared cluster. Because segmentation results are also important for quality control, a number of intermediate data are also released.

### Data, intermediaries, and limitations

Since nuclear segmentation provides the basis for morphometric analysis, subtyping, and survival analysis, it is being released for visualization through our web site at http://tcga.lbl.gov, where users can pan and zoom through the images and overlay segmentation results on original images. The web site also enables exclusion of specific tissue sections for subtyping and genomic association. Computed representations and subtyping is also released through our web site to the community.

**Figure 5:**
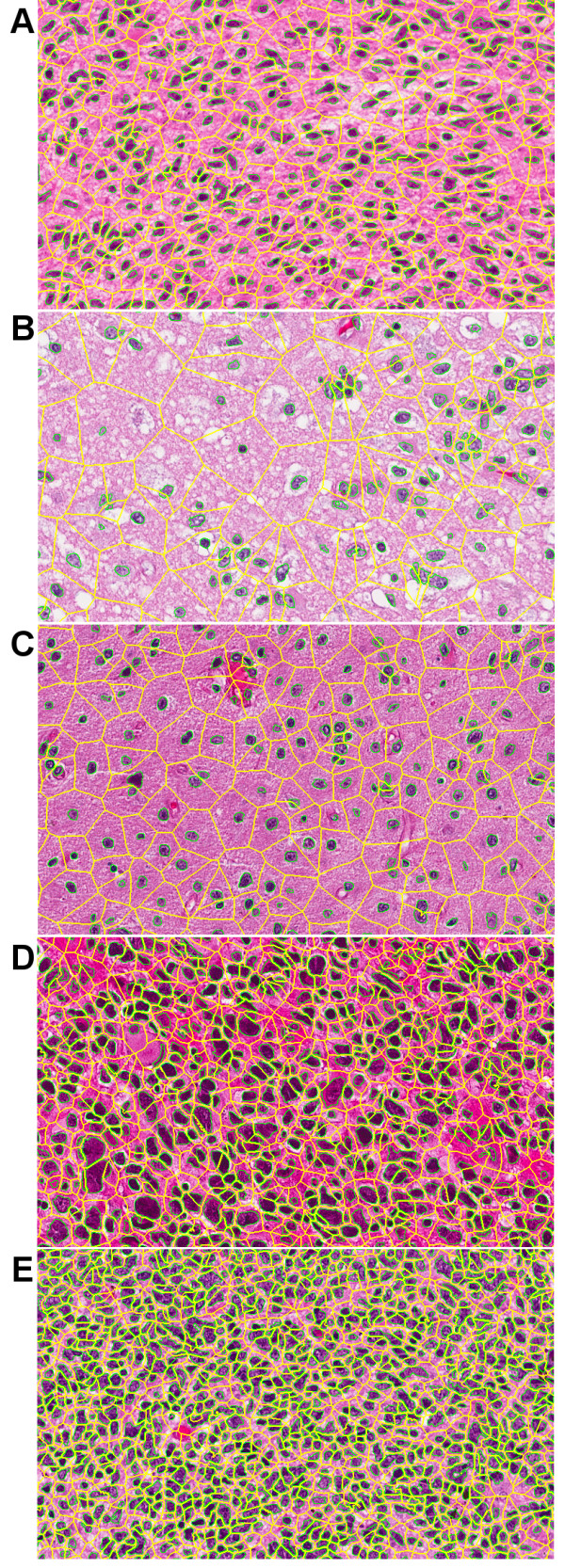
**Nuclear segmentation and region-based tessellation for preferred subtypes of Figure 6E: (A) high cellularity, (B) low cellularity, (C) medium cellularity, (D) high cellularity with pleomorphism, and (E) extreme high cellularity**.

Present limitations are absence of (i) an improved nuclear segmentation method, (ii) patch-based tissue-based labeling, (iii) a systematic evaluation of the multidimensional representation, as it relates to the underlying biology, and (iv) abstraction and complete automation in the computational pipeline. (i) Like others, our approach to nuclear segmentation is not perfect and introduces morphometric errors. The major limitation for introducing more powerful algorithms has been limited computational time for processing very large sets of data. However, given a very large number of cells in a tissue section, subsequent consensus-based clustering tends to treat segmentation errors as outliers. Figure 5 shows nuclear segmentation and region-based tessellation overlaid on images with diverse morphometric signatures, where the cellularity index (e.g., density of cells in a region) is computed as the inverse of each tessellated region area and its density distribution. (ii) In certain tumor types, nuclear segmentation is insufficient for characterizing tissue histopathology. For example, in GBM, apoptotic and necrotic rates are also important. There are also patches where the state of the tissue is transitory, i.e., both apoptotic and necrotic states coexist in a population of cells. This is a higher level of analysis that is difficult to deduce from a simple nuclear segmentation and additional prior knowledge is needed. (iii) Over 50 features are computed per cell, and we have only begun to evaluate some of those that correlate with the known pathology (e.g., nuclear size, cellularity). It is desirable to have an informatics layer for formulating a query and get a different view of the data. Possible use-cases are dimensionality reduction (e.g., PCA, MDS), or feature selection based on outcome or known pathology that is followed by subtyping. Each of these queries provide a unique insight and into the underlying biology for hypothesis generation. (iv) Ideally, all processes should be launched, monitored, and validated through the database. Although, images and computed features are registered with the database, additional queries and notification services are required to construct a more flexible system as required in items (ii) and (iii).

### Quality control

Three modules are tested in the computational pipeline: (i) segmentation, (ii) feature extraction, and (iii) subtyping. (i) We have created a subset of hand segmented images, which originate from a diverse set of tissue sections from TCGA GBM dataset. Even though most images are stained properly, the emphasis on this subset has been placed on blocks where the nuclear dye is heterogeneous. The recall and precision is at 78% and 65%, respectively; (ii) feature extraction and representation were tested against synthetic data with known ground truth; (iii) subtyping is evaluated qualitatively by displaying group similarity matrix.

### Subtyping based tissue histology and survival analysis

**Figure 6:**
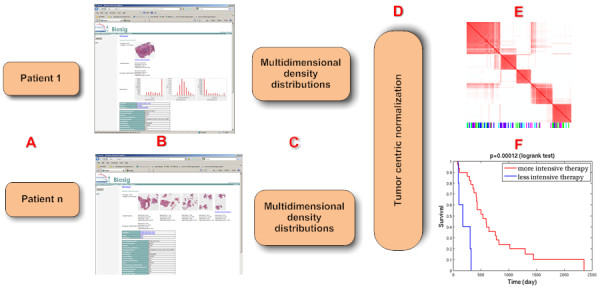
**Steps in identifying subtypes from morphological descriptors of a tissue section**. (A) Each patient may have multiple tissue sections, which are accessible along with the computed features and coded clinical information through BioSig in (B). (C) Each feature, from each tissue, is represented as a density distribution that is normalized in (D). (E) Subtyping identifies 5 classes through consensus voting. (F) Following the Kaplan Meier test, only one subtype proved to have a significant p-value between pair-wise survival curves.

Our system represents each nucleus as a multidimensional vector in the tissue section. We have opted the policy to allow the pathologist to explore clinical questions in terms of selected morphometric indices. This is based on the fact that each morphometric feature can represent underlying biological processes. For example, when the cells are stressed, macromolecules are excreted into cytoplasm (or ECM) to create a textured topography as opposed to a smooth one in normal cells. In the following experiment, it was decided to investigate nuclear size and cellularity for subtyping, survival analysis, and genomic association. The rationale is clear given larger nuclear size and higher proliferation rate in tumor regions. In this experimental configuration, consensus voting revealed five subtypes through qualitative analysis and ordering of the computed similarity matrix, as shown in Figure 5. With respect to correlation with the outcome as a result of therapy, we analyzed patients that received more (e.g., concurrent radiation and chemotherapy or greater than 4 cycles of chemotherapy) or less (e.g., non-concurrent radiation and chemotherapy or less than 4 cycles of chemotherapy) [57]. Following the Kaplan Meier estimator, our analysis indicates that only one subtype with extreme high cellularity, shown in Figure 5E, has a significant p-value through pair-wise comparison of the survival curves using a log-rank test [58]. Figure 6F indicates that with a more intensive therapy (the red curve) life span is increased as compared to a less intensive therapy (blue line). The p-values of other subtypes were not favorable for survival analysis. A possible interpretation is that extreme high cellularity is more homogeneous and highly proliferative; thus, responding better to a more aggressive therapy.

### Genomic association

Given that the therapeutic regime has increased life span for the subtype with extreme high cellularity, as shown in Figure 5E, we queried for its molecular marker through differential gene expression analysis as well as random forest. Both gene lists are provided in Additional file 1, and a more detailed discussion of the gene lists through random forest follows. We have analyzed the top 100 genes for pathway and subnetwork enrichment analysis through *Pathway Studio*. Pathway analysis reveals enrichment of immune response, such as NK-cell (Natural killer cell) and T-cell activation, and AP-1 signaling with p-value of less than 0.05. In support of these findings, the literature suggests that GBM expresses antigens that is recognized by the immune system to eliminate virus infected cells and GBMs [59,60]. Tumor associated antigen (TAA) indicates that glioma cells can be recognized by the immune response, but this process is hindered by the tumor location and evasion strategies developed by GBM. AP-1 (JUN oncogene) is a transcription factor is responsible for high level regulation of IL-13Ra2 that is expressed in GBM cells [61], and is also a highly ranked gene in the TCGA gene tracker.

**Figure 7:**
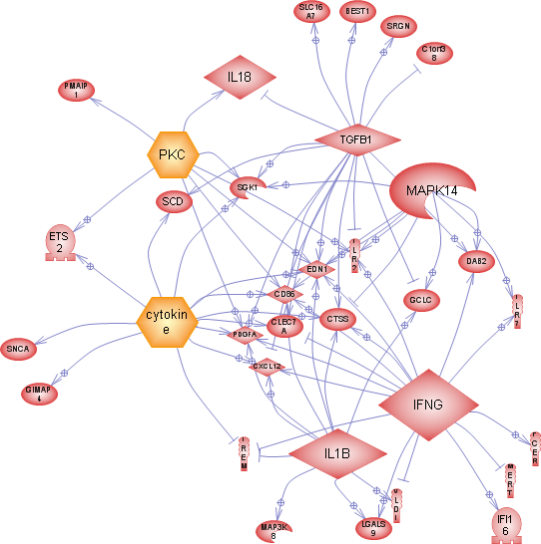
**Subnetwork enrichment analysis has revealed 6 hubs with p-value < 0.05: IFNG, TGFB1, MAPK14, Cytokine, PKC, and ILB1**. Union of these subnetworks and interactions indicates interactions between of these hubs.

Subnetwork enrichment analysis has revealed six hubs, with p-values of less than 0.05 that regulate eight or more other components. These are *IFNG, TGFB1, MAPK14*, Cytokines, *PKC*, and *IL1B*. The union of these subnetworks is shown in Figure 7. *IFNG *and *MAPK14 *are shown to be highly scored by TCGA gene tracker; *TGFB1 *is known to be upregulated in GBM [62]; *PKC *(Protein Kinase C) is well established in cancer signaling and therapy as it is involved in proliferation, migration, and malignant transformation [63], and its isozyme has been suggested for chemotherapeutic targets in GBM [64]; and *ILB1 *is down stream of *NF-kB *and is known to play an important role in cellular response to stress [65] and is constitutively activated in most tumor types. In summary, bioinformatics analysis has provided hypotheses for new modes of potential therapy based on morphometric subtyping.

### Comparison with prior art

It is important to note that another laboratory [66] has analyzed the same dataset. There are differences in the outcome and methodologies. For example, they have reported four subtypes in the GBM dataset. We suggest that the (i) addition of the cellularity index, (ii) utility of feature distributions as opposed to the feature means, (iii) selection of specific combination of features as opposed to all computed features, and (iv) absence of curation have been the deciding factors. Besides cell-based multidimensional representation, there are also differences in nuclear segmentation. It is difficult to assess the differences in segmentation in the absence of source code and computed results on a large dataset; however, color normalization (with respect to the gold standard) and separation of the touching nuclei has not been addressed in [66]. These differences, especially curation, can have a significant impact on morphometric analysis. Finally, we have designed and built an open system, where algorithms and software are going through constant improvement, and computed representation and intermediaries are being made available for each version of the software.

## Conclusions

We have developed an integrated pipeline to process large scale tissue sections for morphometric analysis. The data are downloaded from the NIH web site, partitioned into blocks, and then processed on a cluster. Computed representation is then transferred to a database where (i) data can be downloaded for molecular association, and (ii) computed information is overlaid on the original image and that through panning and zooming, quality control can be performed. Thus far, GBM and kidney data have become publicly available.

We have shown that through morphometric analysis and cellular organization of tissue histology of a large dataset, subtypes can be identified that are predictive of outcome as a result of therapeutic protocol. The main theme is that histological subtyping reveals intrinsic categories that are independent of supervised histological grades. In other words, TCGA's large curated dataset offers potential for revealing subtypes based on *intrinsic *properties of tissue signatures as opposed to the classical tumor grading (e.g., Gleason ranking in prostate cancer), practiced by pathologists. In this context, TCGA's histology database can provide a complementary repository for diagnostic and molecular underpinning for histological subtypes. Subsequently, molecular signature of a subtype can hypothesize a more effective targeted therapy. Our continued research focuses on addressing limitations that has been addressed in the Result section. Ultimately, we plan to develop a system that will process all tumor types.

## Authors' contributions

HC developed high performance software for segmentation and feature-based representation. GVF developed integrated computational pipeline for routing data between NIH repository, local databases, and cluster computing. JH performed subtyping and bioinformatics analysis of multidimensional representation. GC developed the web pages for data distribution and image zooming. FB is the lead pathologist who contributed to the interpretation of subtyping results. JG and PS conceived the study and requirements. BP contributed to the study and experimental plan, led the research, and wrote the paper. All authors read and approved the final manuscript.

## Supplementary Material

Additional file 1 **Supplementary Material for Morphometic Analysis of TCGA Glioblastoma Multiforme**. Supplementary Material for Morphometic Analysis of TCGA Glioblastoma Multiforme. Click here for file
