# Supplementary material for: Morphometic analysis of TCGA glioblastoma multiforme
Source: BMC Bioinformatics. 2011 Dec 20;12:484. doi: 10.1186/1471-2105-12-484 (PMC3271112; doi:10.1186/1471-2105-12-484)
Supplement: Additional file 1 — Supplementary Material for Morphometic Analysis of TCGA Glioblastoma Multiforme. Supplementary Material for Morphometic Analysis of TCGA Glioblastoma Multiforme. [file 1471-2105-12-484-S1.PDF]

Supplementary Material for Morphometic Analysis of TCGA Glioblastoma Multiforme

Hang Chang, Gerald V. Fontenay, Ju Han, Ge Cong, Frederick L. Baehner, Joe W. Gray, Paul T. Spellman, and Bahram Parvin

1. Morphometric features

A number of cellular features are computed through the computational pipeline. The details are summarized in Table S1.

Table S1. Summary of cellular features.

Feature type	Feature name	Annotation
Cellular organization	cell_voronoi_area	
Cellular organization	cellularity	cell density per unit area, inverse of cell_voronoi_area
Cellular organization	edge_length	
Cytoplasm feature	background_intensity_mean	
Cytoplasm feature	background_intensity_sd	sd – standard deviation
Cytoplasm feature	contrast_mean	
Cytoplasm feature	gradient_mean	
Cytoplasm feature	gradient_sd	
Cytoplasm feature	intensity_mean	
Cytoplasm feature	intensity_sd	
Cytoplasm feature	intensity_total	
Nuclear feature	area	
Nuclear feature	background_intensity_mean	
Nuclear feature	background_intensity_sd	
Nuclear feature	bending_energy_s1_mean	
Nuclear feature	contrast_mean	
Nuclear feature	curvature_s1_sd	
Nuclear feature	deviation_from_polygon_convexity	
Nuclear feature	gradient_mean	
Nuclear feature	gradient_sd	
Nuclear feature	intensity_mean	
Nuclear feature	intensity_sd	
Nuclear feature	intensity_total	
Nuclear feature	major_axis	
Nuclear feature	max_curvature_s1	
Nuclear feature	minor_axis	
Nuclear feature	orientation	
Nuclear feature	perimeter	
Nuclear feature	texture_feature_0_mean	1st order steerable filter at angle 0
Nuclear feature	texture_feature_0_sd	1st order steerable filter at angle 0
Nuclear feature	texture_feature_1_mean	2nd order steerable filter at angle 0
Nuclear feature	texture_feature_1_sd	2nd order steerable filter at angle 0
Nuclear feature	texture_feature_2_mean	3rd order steerable filter at angle 0
Nuclear feature	texture_feature_2_sd	3rd order steerable filter at angle 0
Nuclear feature	texture_feature_3_mean	1st order steerable filter at angle 0.78
Nuclear feature	texture_feature_3_sd	1st order steerable filter at angle 0.78

Nuclear feature	texture_feature_4_mean	2nd order steerable filter at angle 0.78
Nuclear feature	texture_feature_4_sd	2nd order steerable filter at angle 0.78
Nuclear feature	texture_feature_5_mean	3rd order steerable filter at angle 0.78
Nuclear feature	texture_feature_5_sd	3rd order steerable filter at angle 0.78
Nuclear feature	texture_feature_6_mean	1st order steerable filter at angle 1.56
Nuclear feature	texture_feature_6_sd	1st order steerable filter at angle 1.56
Nuclear feature	texture_feature_7_mean	2nd order steerable filter at angle 1.56
Nuclear feature	texture_feature_7_sd	2nd order steerable filter at angle 1.56
Nuclear feature	texture_feature_8_mean	3rd order steerable filter at angle 1.56
Nuclear feature	texture_feature_8_sd	3rd order steerable filter at angle 1.56
Nuclear feature	texture_feature_9_mean	1st order steerable filter at angle 2.34
Nuclear feature	texture_feature_9_sd	1st order steerable filter at angle 2.34
Nuclear feature	texture_feature_10_mean	2nd order steerable filter at angle 2.34
Nuclear feature	texture_feature_10_sd	2nd order steerable filter at angle 2.34
Nuclear feature	texture_feature_11_mean	3rd order steerable filter at angle 2.34
Nuclear feature	texture_feature_11_sd	3rd order steerable filter at angle 2.34

2. Genomic association results

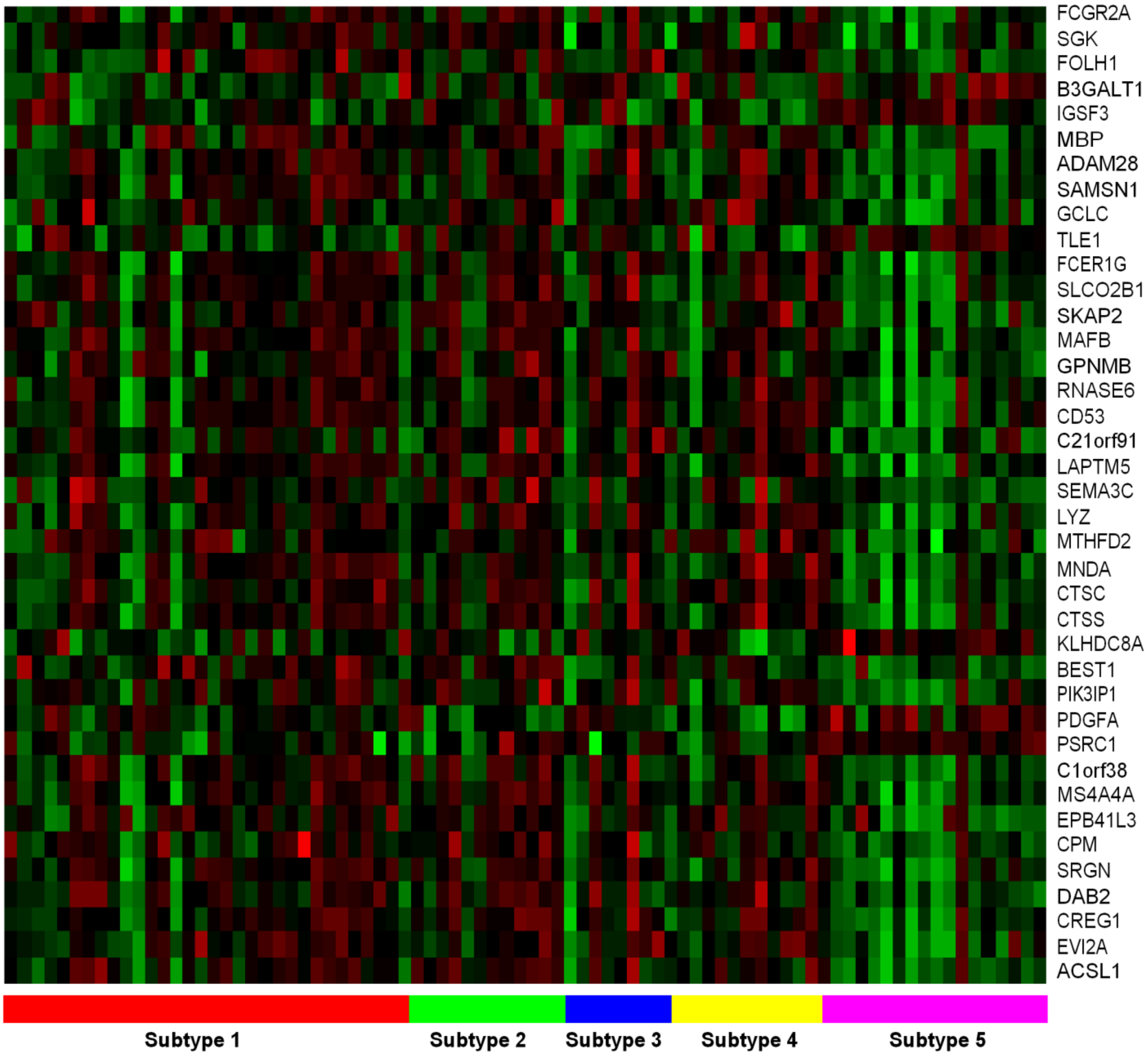

Figure S1. Heatmap of top genes that are differently expressed between the subtype with extremely high cellularity and other subtypes (with FDR adjusted p-value, i.e., q-value, less than 0.06).

Table S2. Top genes that are differently expressed between the subtype with extremely high cellularity and other subtypes.

GeneSymbol	pvalue	qvalue	logFC
ACSL1	4.18E-05	0.032114	-0.82725
EVI2A	6.45E-05	0.032114	-1.34608
CREG1	8.11E-05	0.032114	-0.88965
DAB2	0.000175	0.048859	-0.83521
SRGN	0.000206	0.048859	-1.01384
CPM	0.000337	0.056022	-0.98668
EPB41L3	0.000356	0.056022	-0.94388

MS4A4A	0.000514	0.056022	-1.311
C1orf38	0.000569	0.056022	-0.72694
PSRC1	0.000571	0.056022	0.659637
PDGFA	0.000581	0.056022	0.971521
PIK3IP1	0.000657	0.056022	-0.49174
BEST1	0.000657	0.056022	-0.49247
KLHDC8A	0.000738	0.056022	0.820523
CTSS	0.000741	0.056022	-0.77563
CTSC	0.000814	0.056022	-0.75733
MNDA	0.000845	0.056022	-0.89662
MTHFD2	0.000848	0.056022	-0.60528
LYZ	0.000923	0.057753	-1.10961
SEMA3C	0.001021	0.05912	-1.07837
LAPTM5	0.001053	0.05912	-0.83005
C21orf91	0.001164	0.05912	-0.70219
CD53	0.001251	0.05912	-0.98921
RNASE6	0.001271	0.05912	-0.89634
GPNMB	0.001326	0.05912	-1.3118
MAFB	0.001375	0.05912	-0.92721
SKAP2	0.001502	0.05912	-1.05174
SLCO2B1	0.001609	0.05912	-0.62887
FCER1G	0.001617	0.05912	-0.897
TLE1	0.001622	0.05912	0.529179
GCLC	0.001655	0.05912	-0.5216
SAMSN1	0.001656	0.05912	-0.89771
ADAM28	0.001727	0.05912	-0.56721
MBP	0.001776	0.05912	-0.98078
IGSF3	0.001803	0.05912	0.612816
B3GALT1	0.001836	0.05912	0.629483
FOLH1	0.001841	0.05912	-0.68078
SGK	0.001915	0.059807	-0.68398
FCGR2A	0.001963	0.059807	-0.7478
CNGA3	0.002107	0.06017	0.812964
LCP1	0.002129	0.06017	-0.64423
RNASE1	0.002135	0.06017	-0.73772
KLHL9	0.002177	0.06017	-1.03095
KIAA1598	0.002304	0.060831	-0.90783
ENPP4	0.00237	0.060831	-0.80502
ARHGDIB	0.002484	0.060831	-0.57243
LRRC16	0.002496	0.060831	0.731541
ENPP2	0.002497	0.060831	-1.0113
MS4A6A	0.00255	0.060831	-0.78368
ETS2	0.002559	0.060831	-0.50179
GNAI1	0.002664	0.061401	-0.76828

TLR2	0.002687	0.061401	-0.71847
PLEK	0.00285	0.063898	-0.6305
LCP2	0.002908	0.06401	-0.61621
CDR1	0.003079	0.065219	-0.98837
DOCK2	0.00308	0.065219	-0.61036
AIF1	0.003138	0.065219	-0.71557
C3AR1	0.003185	0.065219	-0.76848
TYROBP	0.003238	0.065219	-0.8076
MAP3K8	0.00363	0.071897	-0.54701
LIPA	0.003773	0.073399	-0.62531
MX2	0.00389	0.073399	-0.64912
PTPRC	0.003929	0.073399	-0.71516
LGALS9	0.00398	0.073399	-0.62286
PAX6	0.004025	0.073399	0.596284
POPDC3	0.004178	0.073399	-0.84053
SELL	0.004256	0.073399	-0.54574
HPSE	0.004265	0.073399	-0.71921
TNS3	0.00433	0.073399	-0.51046
PSCDBP	0.004343	0.073399	-0.63091
FOLR2	0.004433	0.073399	-0.73019
CKB	0.004447	0.073399	0.922206
FYB	0.004601	0.074135	-0.68655
CD37	0.004616	0.074135	-0.55824
ADORA3	0.004745	0.074571	-0.65305
DYNC1I1	0.004769	0.074571	-0.88906
UGDH	0.004897	0.074863	-0.43561
IFI16	0.004964	0.074863	-0.83324
UCP2	0.005001	0.074863	-0.58105
SPON1	0.005056	0.074863	0.969617
SOCS2	0.005102	0.074863	0.899967
ID4	0.005169	0.074915	0.719291
NELL2	0.005304	0.075096	0.855923
HLA-DMB	0.005368	0.075096	-0.67176
C1QB	0.005384	0.075096	-0.68427
LY86	0.005715	0.075096	-0.81574
GYPC	0.005793	0.075096	-0.52554
HCLS1	0.005828	0.075096	-0.73442
VSIG4	0.005871	0.075096	-0.97639
EVI2B	0.006018	0.075096	-0.63395
HSD17B11	0.006099	0.075096	-0.43949
PIP4K2A	0.006143	0.075096	-0.47736
KCTD14	0.006182	0.075096	0.563483
SLC15A3	0.006224	0.075096	-0.48641
LAIR1	0.006282	0.075096	-0.56333

TLR4	0.006284	0.075096	-0.60916
HLA-DPA1	0.006324	0.075096	-0.62512
RAC2	0.006398	0.075096	-0.46807
SCPEP1	0.006472	0.075096	-0.48623
DENND2A	0.006527	0.075096	0.572979

Table S3. Top genes that best predict the subtype with extremely high cellularity through Random Forest analysis.

GeneSymbol	Importance
EPB41L3	0.002202
PIK3IP1	0.001198
ACSL1	0.001098
ENPP4	0.001086
COLEC12	0.001064
C21orf91	0.000895
EVI2A	0.000868
DENND2A	0.000745
DAB2	0.000682
ARNTL	0.000649
BEST1	0.000643
CREG1	0.000638
PDGFA	0.000621
AIM2	0.000616
CDR1	0.000571
CXCL12	0.000557
TTYH1	0.000544
MTHFD2	0.000543
PTER	0.000496
ST3GAL6	0.000495
PSRC1	0.000452
RNASE1	0.000403
SRGN	0.000396
TSPAN7	0.000381
RNASE6	0.000373
ADAM28	0.000373
IFI30	0.000363
PTPRM	0.000361
CKB	0.000361
TPP1	0.000343
SULF1	0.000342
IL18	0.000339
CHML	0.000327
SPOCK1	0.000322
SLC15A3	0.000313
CP110	0.000313

CD53	0.000307
VLDLR	0.000306
GIMAP4	0.000299
TCN2	0.000297
GCLC	0.000287
SKAP2	0.000278
HIP1	0.000274
NFE2L3	0.000267
RASGRP3	0.000262
KLHL9	0.000262
SGK	0.000262
TREM2	0.000255
CAPN3	0.000254
ETS2	0.000252
NLGN4X	0.000251
RNASEH2A	0.000242
ZNF536	0.000241
CPM	0.000241
PIP4K2A	0.000239
C1orf38	0.000236
DTYMK	0.000225
SLC16A7	0.000223
TNS3	0.000219
PMAIP1	0.000213
EDN1	0.000212
LAPTM5	0.000212
RAB4A	0.000212
DDX25	0.00021
DOCK2	0.000209
IFI16	0.000209
MX2	0.000208
IGSF3	0.000207
TLR7	0.000204
KIF1A	0.000203
SNCA	0.000202
IQGAP1	0.0002
ARNTL2	0.000198
PPA1	0.000196
CD86	0.000193
ARL4A	0.000193
MEST	0.000193
EVI2B	0.000193
LCP1	0.000191
MAP3K8	0.000189

DCHS1	0.000188
TLE1	0.000186
ITGB8	0.000183
MERTK	0.000183
DMXL2	0.000183
CTSS	0.00018
FOLH1	0.000179
CLEC4A	0.000175
LCP2	0.000173
CTSC	0.000171
SCD	0.00017
DPY19L1	0.000167
RNASET2	0.000164
FCER1G	0.000162
CLEC7A	0.000161
HSD17B8	0.00016
TLR2	0.000159
LGALS9	0.000158
EPB41L2	0.000156
KLHDC8A	0.000153
